# C-terminal processing of GlyGly-CTERM containing proteins by rhombosortase in *Vibrio cholerae*

**DOI:** 10.1371/journal.ppat.1007341

**Published:** 2018-10-23

**Authors:** Shilpa Gadwal, Tanya L. Johnson, Henriette Remmer, Maria Sandkvist

**Affiliations:** 1 Department of Microbiology and Immunology, University of Michigan Medical School, Ann Arbor, MI, United States of America; 2 Department of Chemistry, Eastern Michigan University, Ypsilanti, MI, United States of America; 3 Department of Biological Chemistry, University of Michigan Medical School, Ann Arbor, MI, United States of America; Northwestern University, Feinberg School of Medicine, UNITED STATES

## Abstract

*Vibrio cholerae* and a subset of other Gram-negative bacteria, including *Acinetobacter baumannii*, express proteins with a C-terminal tripartite domain called GlyGly-CTERM, which consists of a motif rich in glycines and serines, followed by a hydrophobic region and positively charged residues. Here we show that VesB, a *V*. *cholerae* serine protease, requires the GlyGly-CTERM domain, the intramembrane rhomboid-like protease rhombosortase, and the type II secretion system (T2SS) for localization at the cell surface. VesB is cleaved by rhombosortase to expose the second glycine residue of the GlyGly-CTERM motif, which is then conjugated to a glycerophosphoethanolamine-containing moiety prior to engagement with the T2SS and outer membrane translocation. In support of this, VesB accumulates intracellularly in the absence of the T2SS, and surface-associated VesB activity is no longer detected when the rhombosortase gene is inactivated. In turn, when VesB is expressed without an intact GlyGly-CTERM domain, VesB is released to the extracellular milieu by the T2SS and does not accumulate on the cell surface. Collectively, our findings suggest that the posttranslational modification of the GlyGly-CTERM domain is essential for cell surface localization of VesB and other proteins expressed with this tripartite extension.

## Author summary

The type II secretion system (T2SS) critically contributes to pathogenesis of many Gram-negative bacteria by transporting toxins and a variety of enzymes including proteases, lipases, nucleases and carbohydrases to the extracellular environment. Cholera toxin, the principal virulence factor of *Vibrio cholerae*, is an example of a T2SS substrate that employs this multi-protein complex for translocation across the outer membrane. While cholera toxin freely diffuses away from the bacterial cell following outer membrane translocation, other T2SS substrates remain surface associated or may reattach to the bacterial cell surface following extracellular release. We show here that a large fraction of VesB, another *V*. *cholerae* T2SS substrate, stays associated with the cells and that this depends on the posttranslational modification of its C-terminal domain, GlyGly-CTERM, by rhombosortase. Rhombosortase is a member of the nearly ubiquitous rhomboid family of intramembrane serine proteases. As the *Providencia stuartii* TatA is the only rhomboid protease substrate identified in bacteria to date, this study expands the list of known bacterial rhomboid protease substrates and likely includes all proteins expressed with GlyGly-CTERM extensions.

## Introduction

The type II secretion system (T2SS) is a multi-protein complex used by many Gram-negative bacteria to secrete extracellular proteins [1–4]. Most notably, *Vibrio cholerae*, the causative agent of cholera, uses the T2SS to secrete cholera toxin [5, 6]. Cholera toxin and other T2SS substrates are secreted in a two-step process. First, proteins translocate across the inner membrane via recognition of their signal peptide by the Sec or Tat systems. Then, the secretion intermediates fold, engage with the T2SS and traverse the channel formed by the outer membrane embedded secretin [7–9]. These substrates are then free to diffuse away from the cell.

**Fig 1:**
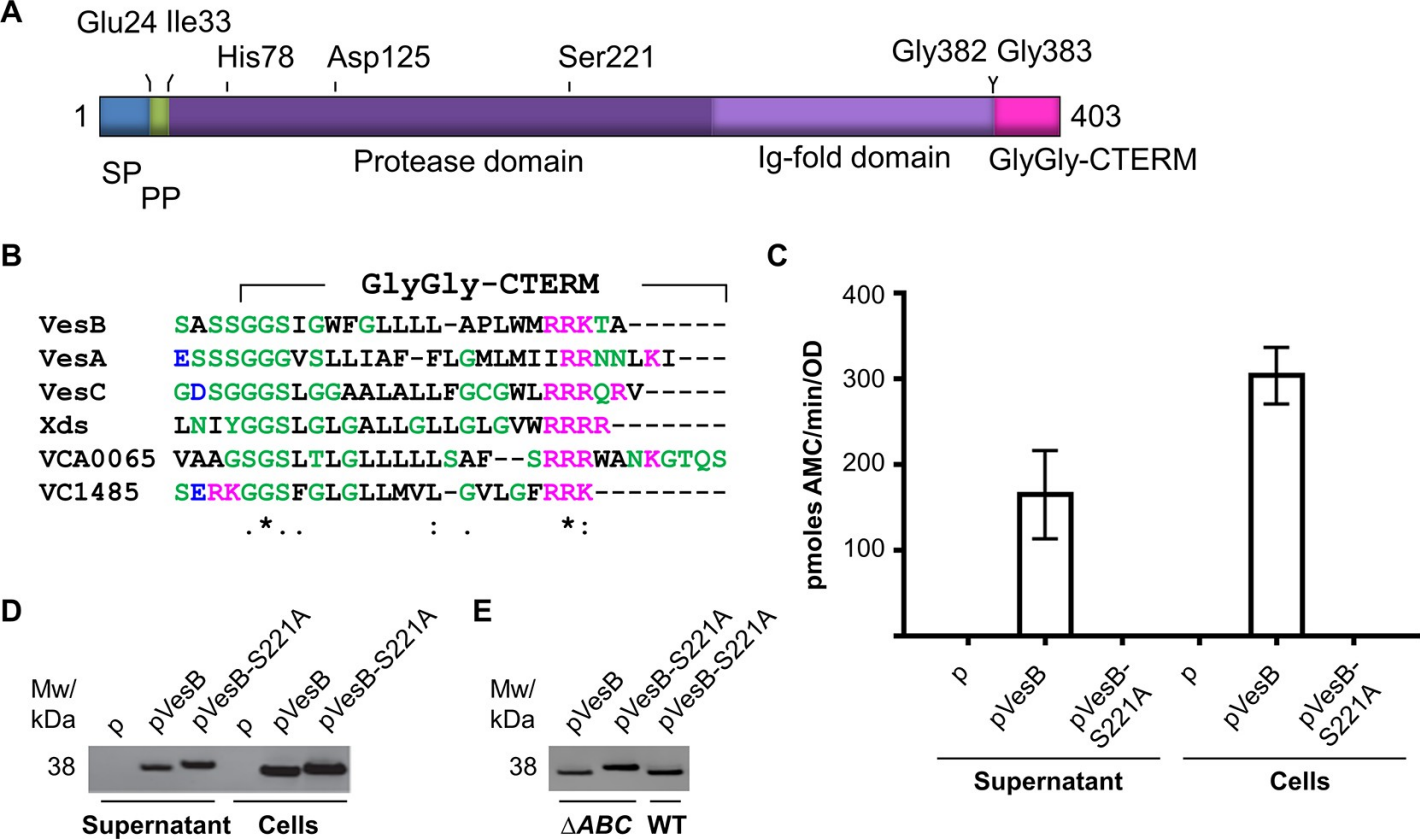
Secreted and cell-associated VesB are active. **A.** Schematic representation of prepro-VesB with the N-terminal signal peptide (SP; residues 1–23) in blue, the pro-peptide (PP; residues 24–32) in green, the protease domain (with the catalytic residues His, Asp and Ser) in dark purple, the Ig-fold domain in light purple, and the GlyGly-CTERM extension (382–403) in magenta. **B.** The primary sequences of the C-terminal portions of the six proteins in *V*. *cholerae* containing the GlyGly-CTERM domain are aligned using the EMBL-EBI multiple sequence alignment tool, Clustal Omega (http://www.ebi.ac.uk/Tools/msa/clustalo/). Residues are labeled according to their physiochemical properties. Magenta represents basic residues. Black denotes small and hydrophobic residues, including aromatic amino acids (except tyrosine). Green residues include glycine and amino acids with either hydroxyl-, sulfhydryl-, or amine groups. Aspartic and glutamic acid are shown in blue. **C.** The *ΔvesABC* strain containing empty vector (p), pVesB, or pVesB-S221A were grown in LB with 100 μg/mL carbenicillin and 50 μM IPTG for four hours. Serine protease activity against the fluorogenic peptide Boc-Gln-Ala-Arg-AMC was measured in culture supernatants and suspensions of intact cells. Samples from three independent experiments were each analyzed in technical triplicates and the bars represent means +/- standard error. **D.** The culture supernatants and cell suspensions from **C** were subjected to SDS-PAGE and immunoblotting with VesB antibodies. Representative blot is shown. **E.** Culture supernatants of WT and *ΔvesABC* strains of *V*. *cholerae* expressing plasmid-encoded VesB or VesB-S221A grown for four hours in LB with 100 μg/mL carbenicillin and 50 μM IPTG were analyzed by SDS-PAGE and immunoblotting with VesB antibodies. Representative blot is shown.

In addition to cholera toxin, *V*. *cholerae* transports a number of other proteins, including three serine proteases, VesA, VesB and VesC, across the outer membrane via the T2SS [6, 10]. VesB, the focus of this study, is a trypsin-like serine protease that contains an N-terminal signal peptide, a protease domain, and an immunoglobulin-like domain [11] (Fig 1A). The protease domain is 30% identical to trypsin and includes a typical His-Asp-Ser catalytic triad and activation site. VesB is made as a zymogen and cleavage at the activation site (Arg_32_-Ile_33_) results in the removal of the N-terminal propeptide to generate active VesB [11]. VesB, along with five other *V*. *cholerae* proteins, VesC, VesA, Xds, VCA0065, and VC1485 also contain a C-terminal domain called GlyGly-CTERM [12] (Fig 1B). Most, if not all, of these so-called GlyGly-CTERM proteins are hydrolytic enzymes. Proteolytic activities of VesA, VesB and VesC have been verified using low molecular weight fluorogenic peptides [10, 11]. VesA and VesB are expressed and active in intestinal cecal fluid during *V*. *cholerae* colonization of rabbits as determined by an activity-based protein profiling technique [13]. While the range of their natural substrates has yet to be determined, VesA and VesB are capable of cleaving the A subunit of cholera toxin, thus separating the A1 and A2 domains required for toxin activation [10], and VesC induces a hemorrhagic fluid response when injected into rabbit and mouse ileal loops [14, 15]. Xds is the most well characterized of the GlyGly-CTERM containing proteins. Through its nuclease activity Xds generates nutrients, combats neutrophil extracellular traps, and modulates biofilm [16–18]. Lastly, VCA0065 is a RpoH-regulated putative Zn-metalloprotease, while VC1485 is a protein with unknown function that may be essential for growth [19–22].

The novel GlyGly-CTERM domain has a consensus tripartite motif that contains two prominent glycines surrounded by serines, followed by a hydrophobic helix and positively charged residues (Fig 1B). It was originally identified bioinformatically in Gram-negative bacteria of the *Vibrio*, *Shewanella*, *Acinetobacter* and *Ralstonia* genera [12]. Through *in silico* partial phylogenetic profiling, proteins that have a GlyGly-CTERM extension were identified to co-exist with a putative intramembrane protease, rhombosortase, while GlyGly-CTERM proteins are absent in bacteria lacking rhombosortase [12]. In addition, in species encoding only one GlyGly-CTERM protein, the gene is linked to the gene coding for rhombosortase. This co-distribution led the authors to speculate that rhombosortase, if expressed as an active enzyme, may target the C-terminal extension of the GlyGly-CTERM proteins [12]. However, besides this bioinformatics analysis, the relationship between GlyGly-CTERM and rhombosortase has not been experimentally validated.

Rhombosortase is homologous to rhomboid proteases, a class of membrane-embedded serine proteases that cleave single pass transmembrane proteins at or within the plane of the lipid bilayer [23–29]. X-ray crystallography studies of the rhomboid protease GlpG indicate that it is comprised of six transmembrane domains and a cavity within its folded form that contains the catalytic residues Ser and His [23–26]. Rhomboid proteases are nearly ubiquitous and function in a wide range of processes [30–32]. They have been implicated in human and parasitic diseases; however, only one rhomboid protease substrate has been identified thus far in bacteria. The *Providencia stuartii* rhomboid protease AarA cleaves off a short N-terminal peptide and thereby activates the channel-forming TatA of the twin-arginine translocation system [33, 34].

In this study, we address the role of the T2SS, rhombosortase and the GlyGly-CTERM domain in VesB biogenesis. Analysis of the localization and activity of VesB in different mutant backgrounds reveals that rhombosortase cleaves the GlyGly-CTERM domain and promotes T2SS-dependent surface localization of active VesB.

## Results

### Cell-associated VesB is active

Previously, we have shown that while VesB is a type II secreted protease with its N-terminal signal peptide and propeptide removed [10, 11], immunoblotting of culture supernatants and cells indicated that a large fraction of VesB remains cell-associated. To determine whether the cell-associated form is active, we expressed plasmid-encoded VesB in a strain lacking the genes for the three related serine proteases VesA, VesB and VesC (Δ*vesABC* [10]). We included two additional strains, one containing an empty vector and one expressing an active site mutant protein, VesB-S221A, to serve as negative controls. Cultures were grown in the presence of 50 μM IPTG in LB for 4 hours, after which culture supernatants and cells were separated by centrifugation and the VesB activity was determined. When using the fluorogenic peptide Boc-Gln-Ala-Arg-AMC as a substrate, VesB activity was found in the culture supernatant and in association with intact cells (Fig 1C), suggesting that both the secreted and cell-associated forms of VesB are proteolytically active. While VesB-S221A was inactive in this assay, it could be detected in culture supernatant and in association with cells with anti-VesB antibodies (Fig 1D). Like other trypsin-like proteases, VesB is produced as a zymogen and removal of the propeptide through processing at Arg_32_-Ile_33_ results in activation of VesB [11]. The finding that VesB-S221A migrated slower compared to VesB when analyzed by SDS-PAGE and immunoblotting indicated that it still contains the propeptide, suggesting that VesB is an autoactivating enzyme (Fig 1D). Further support for this came from comparing the size of plasmid-expressed VesB-S221A in the absence and presence of native VesB. When co-expressed with native VesB in a wild type (WT) strain background, VesB-S221A migrated on SDS-PAGE indistinguishably from that of WT VesB (Fig 1E).

### VesB is localized to the cell surface

**Fig 2:**
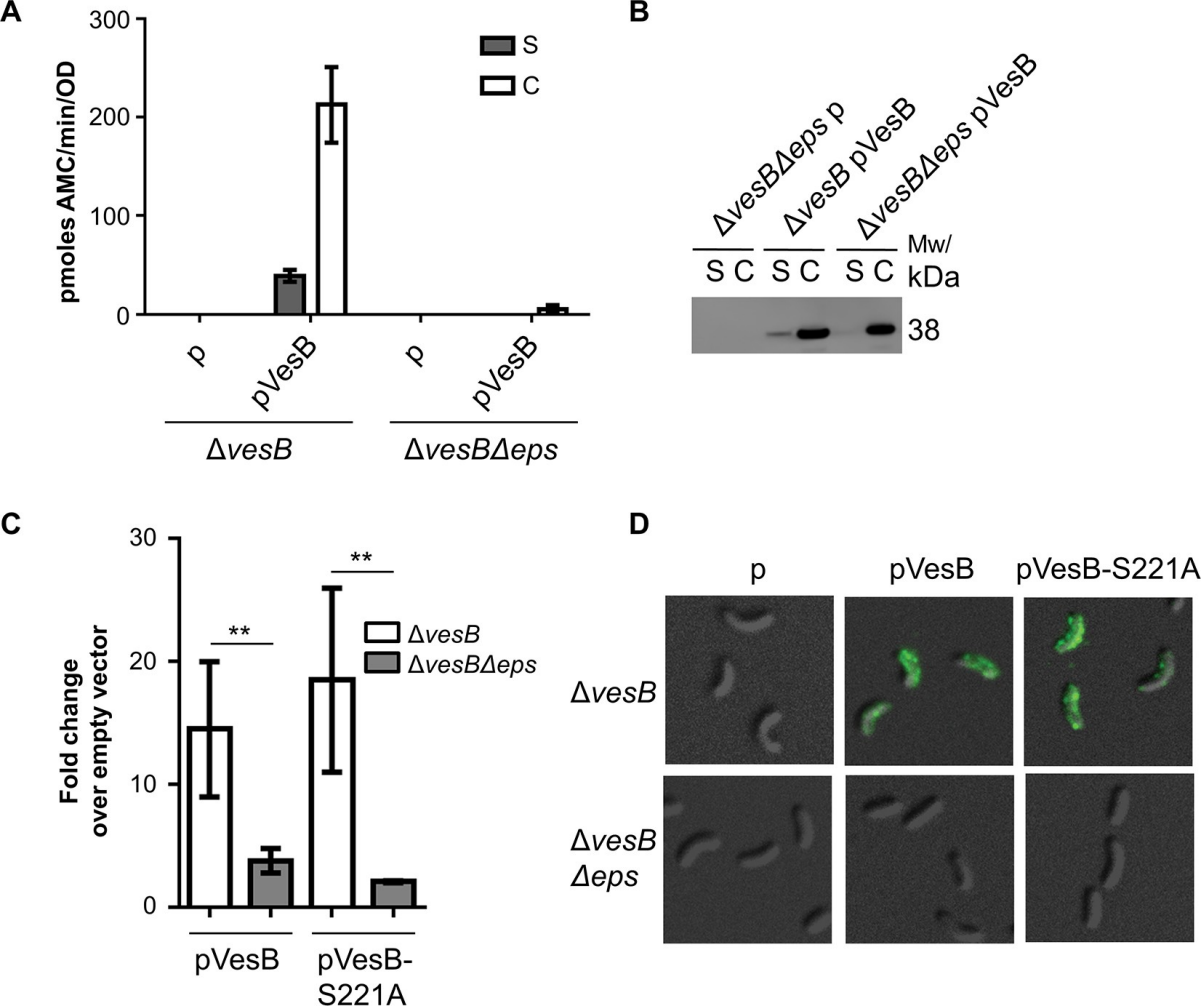
VesB is surface-associated in WT but not in the T2SS mutant. The Δ*vesB* and Δ*vesB*Δ*eps* strains containing empty vector (p) or pVesB were grown for four hours in M9 media supplemented with glucose and casamino acids in the presence of 100 μg/mL carbenicillin and 50 μM IPTG. **A.** Supernatants (S) and whole cells (C) were isolated and analyzed for protease activity using the fluorogenic peptide Boc-Gln-Ala-Arg-AMC. Samples from three independent experiments were each analyzed in technical triplicates. Bars show mean ± S.E. **B.** Samples from A were analyzed by SDS-PAGE and immunoblotting with VesB antibodies. Representative blot of one of three separate experiments. **C.** Cells were washed and incubated with VesB antibodies followed by Alexa Fluor 488 antibodies. The cells were subjected to quantitative fluorimetry and the fluorescence units were normalized by OD_600_ of the original cultures and fold changes over cells with empty vector were calculated. The experiments were done in triplicates and standard error bars are shown. P values were generated by comparing Δ*vesB* and Δ*vesB*Δ*eps* strains containing pVesB or pVesB-S221A (**p<0.01). **D.** Cells that were labeled with VesB antibodies followed by Alexa Fluor 488 antibodies were visualized by differential interference contrast and fluorescence microscopy. Representative images of three independent experiments are shown.

Because we detected cell-associated VesB activity, we hypothesized that VesB may be surface-localized in T2SS competent cells, but remains internally trapped in T2SS mutant cells (Δ*eps*). To test this, we expressed plasmid-encoded VesB in the Δ*vesB* and Δ*vesB*Δ*eps* strains and subjected the cells to surface labeling with anti-VesB antibodies and Alexa Fluor 488 F(ab′)_2_ goat anti-rabbit IgG. The cultures were grown in M9 medium supplemented with glucose and casamino acids to avoid autofluorescence observed with LB grown cells. Under this growth condition, the majority of VesB protein and activity was cell-associated in the ΔvesB/pVesB strain and very little VesB was found in the culture supernatant (Fig 2A & 2B). In contrast, in the Δ*vesB*Δ*eps/*pVesB strain, while the majority of VesB remained cell-associated, it was not active (Fig 2A). Furthermore, the plasmid-encoded VesB migrated slightly slower in the Δ*vesB*Δ*eps* strain than in the Δ*vesB* strain (Fig 2B), suggesting that it may retain the N-terminal propeptide and that zymogen activation of VesB by cleavage of the Arg_32_Ile_33_ bond likely occurs after outer membrane translocation.

For the VesB surface labeling experiment, the Δ*vesB* and Δ*vesB*Δ*eps* strains with empty vector served as negative controls. After thorough washing, the labeling efficiency was quantitated by fluorimetry of cells in suspension and the fluorescent units were normalized to the OD_600_ of the original cultures. The fluorescence measured for Δ*vesB/*pVesB cells was 4.8-fold higher when compared to Δ*vesB*Δ*eps/*pVesB cells, suggesting that VesB is surface-localized in WT cells, while it primarily remains intracellular in the Δ*eps* cells (Fig 2C). The cells were also visualized by fluorescence microscopy (Fig 2D). Quantitative analysis indicated that 94% of Δ*vesB/*pVesB cells were fluorescent, whereas there was no detectable fluorescence observed with Δ*vesB*Δ*eps/*pVesB mutant cells. Similarly, the catalytically inactive VesB-S221A was also localized to the surface of cells with an intact T2SS but remained intracellular in Δ*eps* cells (Fig 2C and 2D). This suggests that activation of VesB is not required for surface localization. Taken together, our results suggest that outer membrane translocation by the T2SS is required for VesB localization to the surface of WT cells, and that following translocation VesB autoactivates.

### Rhombosortase-mediated processing of VesB

**Fig 3:**
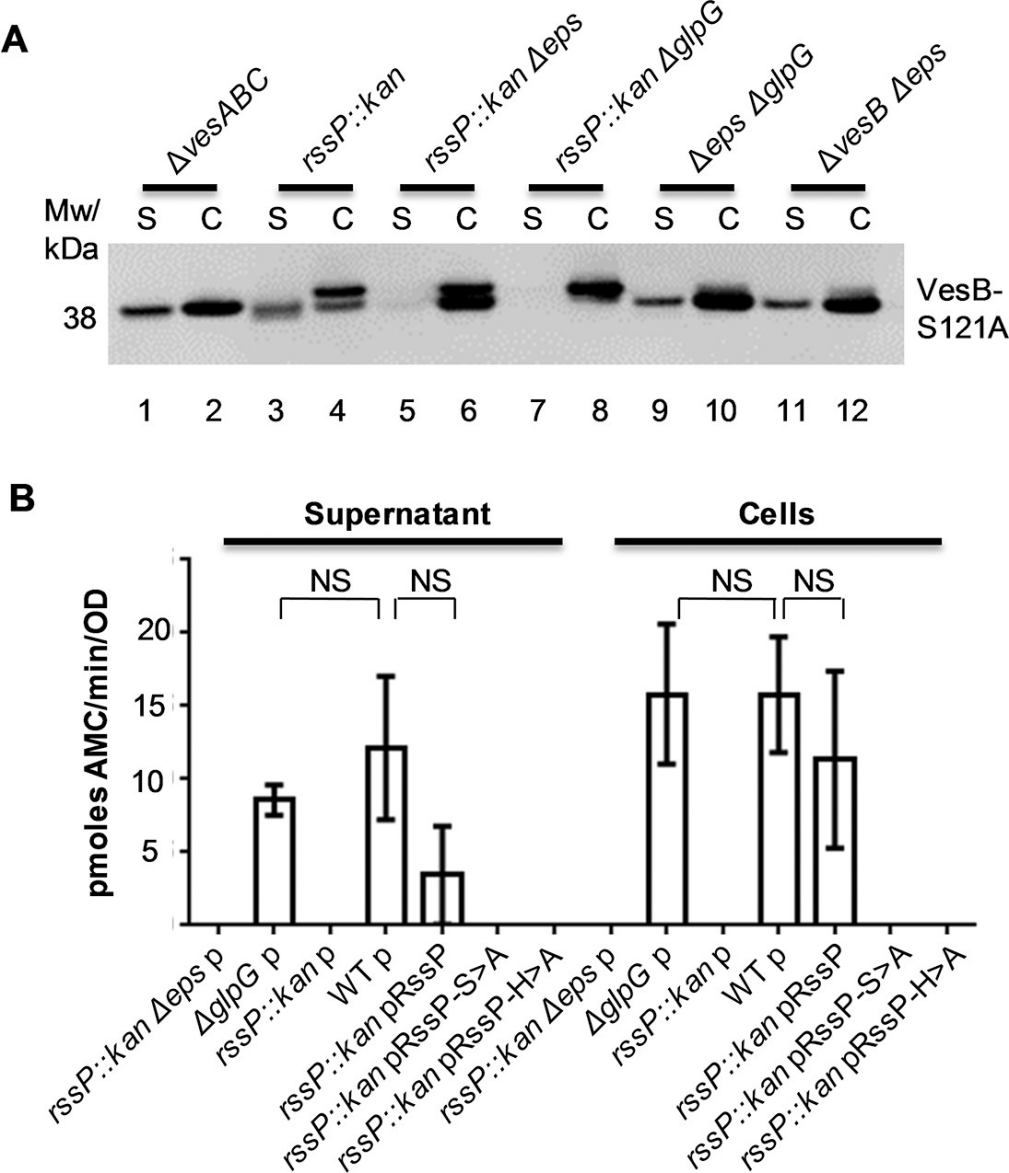
Cleavage of VesB by rhombosortase. **A.** The indicated mutant strains with pVesB-S221A were grown for four hours in M9 media supplemented with casamino acids, glucose, 100 μg/mL carbenicillin and 50 μM IPTG. Culture supernatant (S) and cells (C) were isolated, matched by equivalent OD_600_ units (except for the *ΔvesABC* culture supernatant and the cell samples of the *ΔvesABC*, *ΔglpGΔeps*, and *ΔvesBΔeps* strains which were diluted 1:10) and analyzed by SDS-PAGE and western blotting with VesB antibodies. Representative blot is shown. **B.** WT and the indicated mutants containing empty vector (p), pRssP, pRssP-S102A or pRssP-H160A were grown for 4 hours in LB broth with 100 μg/mL carbenicillin and 10 μM IPTG. Culture supernatants and whole cells were isolated and analyzed for protease activity using the fluorogenic peptide Boc-Gln-Ala-Arg-AMC. The assays were performed on samples from three separate experiments in technical triplicates. The means with corresponding standard errors are shown. The difference in activity of VesB in the WT strain was statistically significant (p<0.01) when compared to the activity of VesB in the other samples except for Δ*glpG*p and *rssp*::*kan* pRssP supernatants and cells (NS).

Through partial phylogenetic profiling, a previous study identified a putative rhomboid protease, rhombosortase, to be co-distributed with proteins containing a GlyGly-CTERM sequence, leading to the speculation that rhombosortase may cleave these proteins [12]. To determine the relationship between VesB and the rhombosortase protease RssP of *V*. *cholerae*, we inactivated the *rssP* gene and subjected culture supernatants and cells to SDS-PAGE and immunoblotting with VesB antibodies. To simplify the analysis and keep the number of processing events to a minimum, we initially used VesB-S221A, which does not undergo autoactivation (Fig 1C and 1D). Culture supernatants and cells were separated following growth of the various mutants in the presence of 50 μM IPTG in M9 media supplemented with glucose and casamino acids, and putative size differences of VesB-S221A were analyzed by SDS-PAGE and immunoblotting (Fig 3A). Because VesB is less stable in *rssP*::*kan* backgrounds it was necessary to load differing amounts of sample from the various strains in order to clearly visualize potential size differences of VesB-S221A. Accordingly, we loaded ten times less of the samples in lanes 1, 2, 10 and 12 (Fig 3A). When the rhombosortase gene was inactivated (*rssP*::*kan*), the total amount of VesB-S221A was reduced and a portion migrated slower than VesB-S221A in cells expressing a functional rhombosortase (Fig 3A, compare lanes 2 and 4). This unprocessed form of VesB-S221A was exclusively detected in the cell fraction (Fig 3A, lane 4). Interestingly, a small amount of VesB-S221A was still cleaved and released extracellularly (Fig 3A, lane 3); however, this cleaved form was no longer released when the T2SS was inactivated in the *rssP*::*kanΔeps* double mutant (Fig 3A, lane 5). While these results suggested that rhombosortase cleaves VesB-S221A, we speculated that the residual processing of VesB-S221A in the *rssP* mutant was due to the activity of the ubiquitous rhomboid protease GlpG, which shares 32% amino acid sequence identity with RssP. To test this, we made a double mutant, where both *glpG* and *rssP* were inactivated (*rssP*::*kanΔglpG)* and found that in this background VesB-S221A remained associated with the cells in an un-processed form (Fig 3A, lane 8), suggesting that GlpG is capable of cleaving VesB-S221A when RssP is absent, albeit inefficiently. As VesB-S221A was completely cleaved in the *ΔepsΔglpG* double mutant (Fig 3A, lanes 9 and 10), it suggests that RssP is the primary protease that cleaves VesB. The results shown in lanes 9–12 also indicate that RssP-mediated processing of VesB does not require the presence of a functional T2SS and suggests that cleavage likely occurs before VesB engages with the T2SS.

### Rhombosortase-mediated processing is a prerequisite for VesB activity

To establish the functional consequences of VesB processing, we next analyzed WT VesB expressed from its native promoter. Specifically, we wanted to determine whether generation of active VesB is dependent on cleavage by rhombosortase. Cultures were grown in LB, and culture supernatants and intact cells were analyzed for VesB activity. In both WT and Δ*glpG* strains, VesB was active in the culture supernatants and in association with cells (Fig 3B), further confirming that GlpG does not play a significant role in VesB biogenesis when rhombosortase is present. In the experiment shown in Fig 3A, VesB-S221A was found to be unstable and detected in two forms when overexpressed in the *rssP*::*kan* mutant and only the GlpG-cleaved form was found in the culture supernatant. Here, when WT VesB was expressed from its native promoter in the *rssP*::*kan* mutant grown in LB no VesB activity was detected (Fig 3B). This is consistent with the earlier finding that VesB is unstable in the absence of rhombosortase, but also suggests that the GlpG-cleaved form of VesB is inactive, and therefore, that the C-terminal cleavage of VesB by rhombosortase is required for the correct maturation of active VesB. Since VesC and VesA collectively contribute to approximately 20% of the proteolytic activity towards Boc-Gln-Ala-Arg-AMC [10], the lack of activity for the *rssP*::*kan* mutant suggests that VesC and VesA are also substrates of rhombosortase.

When the *rssP*::*kan* mutant was complemented with the plasmid pRssP, we observed restoration of protease activity in both the culture supernatant and cells (Fig 3B). In contrast, no complementation of the *rssP*::*kan* mutant was apparent in the presence of pRssP-S102A and pRssP-H160A, which express mutant forms of rhombosortase in which the predicted catalytic residues serine and histidine, respectively, are substituted with alanine (Fig 3B).

### Processing of additional GlyGly-CTERM proteins by rhombosortase

To further analyze the effect of inactivating the *rssP* gene, we took a proteomics approach that involved isolating and concentrating the culture supernatants of the WT and *rssP*::*kan* strains and subjecting them to off-line SDS-PAGE, gel segmentation, in-gel digestion with trypsin, and LC/MS/MS. Many proteins were detected using this method; however, we focused on VesB and the other five GlyGly-CTERM containing proteins. The proteomic data were used to compare the relative amount of the GlyGly-CTERM containing proteins in the culture supernatant of the WT and *rssP*::*kan* strains following growth in LB, a growth condition that supports the expression of all six GlyGly-CTERM proteins. We hypothesized that the amount of the GlyGly-CTERM containing proteins would be reduced in the culture supernatant of the *rssP*::*kan* mutant unless they are cleaved by GlpG, similarly to VesB, in the absence of rhombosortase. Spectral counts for each protein were normalized to total number of spectra in each sample and protein size to obtain Normalized Spectral Abundance Factor (NSAF) values [35] for each protein in the two samples (Table 1). The results showed that the amount of VesA, VesC, Xds, and VCA0065 was significantly reduced in the absence of RssP by four-fold or more (Table 1). Consistent with results from our immunoblotting experiments, VesB was only reduced by a factor of 1.5 in the *rssP*::*kan* mutant, while the level of VC1485 was increased by a factor of 2. Neither of these latter results was statistically significant and is likely due to GlpG’s ability to cleave and thus release VesB, and perhaps also VC1485, to the culture supernatant when rhombosortase is absent.

**Table 1:** Four of the six GlyGly-CTERM containing substrates are present in lower amounts in the supernatant of the rhombosortase mutant.

Protein Name	WT Peptide Coverage (%)	*rssP*::*kan* Peptide Coverage (%)	NSAF WT	NSAF *rssP*::*kan*	WT/*rssP*::kan*
VCA0065	29	20	0.0023	0.0004	5.4
VesC	67	59	0.0127	0.0026	4.9
Xds	33	0	0.0015	0	-
VesA	11	0	0.0015	0	-
VesB	49	50	0.0023	0.0015	1.5
VC1485	59	67	0.0062	0.0125	0.5

*A ratio of ≥ 4 is considered statistically significant. The criteria used for quantitative analysis of data are based on empirical observation of many large datasets. The four-fold change is deliberatively conservative to reduce the number of false positives.

### C-terminal modification of VesB

**Fig 4:**
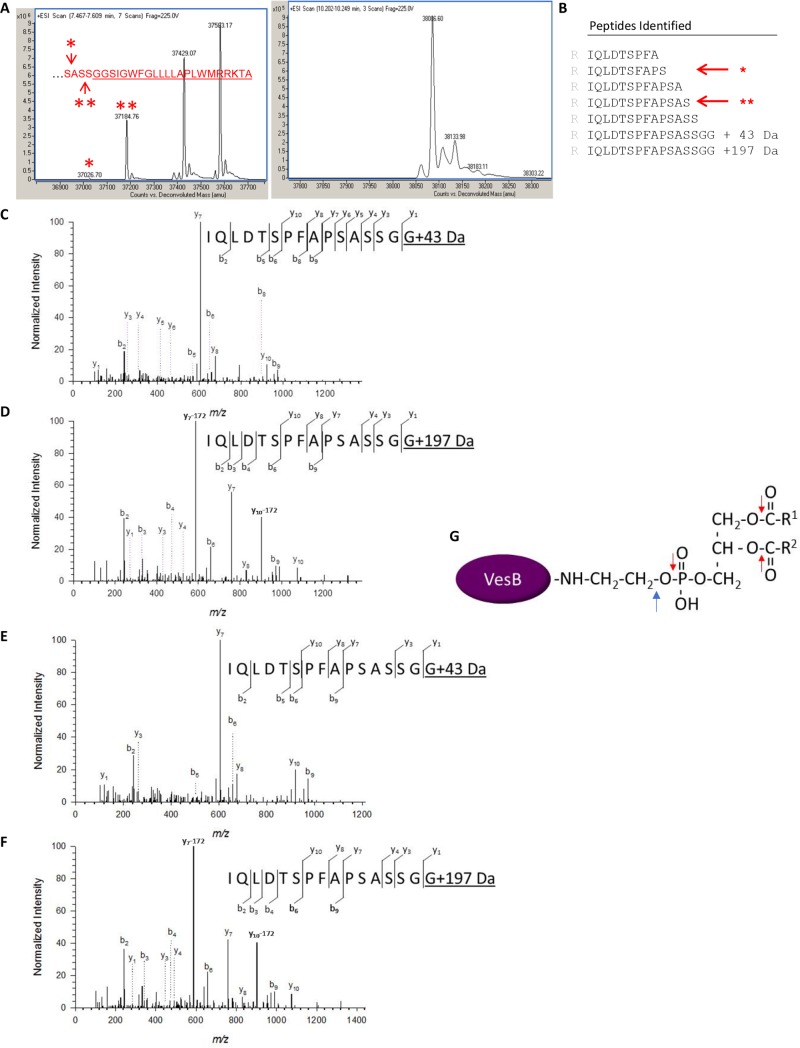
C-terminal modification of VesB. VesB was overexpressed and purified from supernatants of LB-grown *V*. *cholerae* culture in the presence of protease inhibitors at 4° C. The purified material was subjected to intact mass analysis (A) and SDS-PAGE, in-gel trypsin digestion and LC-MS/MS analysis (B-D). **A. Left panel:** Deconvoluted ESI mass spectrum indicates four molecular masses of VesB. The masses highlighted with asterisks correspond to theoretical VesB masses generated through cleavage at either Ser_378_ (_*_) or Ser_380_ (_**_) (see inset with C-terminal sequence of VesB). The relative amount of the four VesB species varied between three separate purifications. Accuracy of the instrument: 0.01% of molecular mass. **Right panel:** The deconvoluted mass spectrum of protein eluting at a higher acetonitrile concentration. **B.** C-terminal peptides of VesB generated by trypsin and identified by LC-MS/MS analysis. The asterisks correspond to cleavage at Ser_378_ (_*_) and Ser_380_ (_**_). **C.** Representative MS/MS spectrum for the C-terminal peptide IQLDTSPFAPSASSGG of VesB shows modification of the C-terminal glycine with a 43 Da moiety as evidenced by the presence of the y1–ion and indicated subsequent y-ions. **D.** MS/MS spectrum for the VesB peptide IQLDTSPFAPSASSGG with a C-terminal 197 Da moiety as evidenced by the presence of the y1–ion and indicated subsequent y-ions as well as the two fragment-ions y_7_-172 at m/z 587.3 and y_10_-172 at m/z 904.2 generated by neutral loss of the phosphoglyceryl moiety (C_3_H_9_O_6_P, 172.013 Da). **E.** VesB was extracted from WT *V*. *cholerae* with Triton X-100, purified on benzamidine sepharose and subjected to SDS-PAGE, in-gel trypsin digestion and LC -MS/MS analysis. Representative MS/MS spectrum of the C-terminal peptide IQLDTSPFAPSASSGG of VesB shows modification of the C-terminal glycine with a 43 Da moiety as evidenced by the presence of the y1–ion and indicated subsequent y-ions. **F.** MS/MS spectrum of the 197-Da modified peptide IQLDTSPFAPSASSGG as evidenced by the presence of the y1–ion and indicated subsequent y-ions as well as the two fragment-ions y_7_-172 at m/z 587.3 and y_10_-172 at m/z 904.2 generated by neutral loss of the phosphoglyceryl moiety (C_3_H_9_O_6_P, 172.013 Da). **G.** VesB is attached to a glycerophosphoethanolamine containing moiety, possibly phosphatidylethanolamine, via its C-terminal glycine. The red and blue arrows indicate possible sites of hydrolysis or fragmentation, respectively, resulting in VesB species with either ethanolamine or glycerophosphoethanolamine.

To identify the rhombosortase cleavage site in VesB and to begin to address the mechanism by which VesB is associated with the surface of *V*. *cholerae*, we subjected both the secreted and surface associated forms of VesB to affinity purification and mass spectrometry analysis. First, we grew *V*. *cholerae* into stationary phase in LB to maximize the yield of overexpressed VesB. Following removal of cells, VesB was purified from the culture supernatant on benzamidine sepharose as described previously [11]. N-terminal sequencing confirmed that the signal peptide and propeptide were removed. Purified VesB was then subjected to reversed phase chromatography and electrospray mass spectrometry (ESI-LC/MS) to obtain its intact molecular mass. Two major peaks representing masses of 37,583 and 37,429 Da were obtained along with two minor peaks representing species of 37,185 Da and 37,027 Da, respectively (Fig 4A; left panel). An additional species, 38,086 Da (Fig 4A; right panel) eluted at a higher percentage of the organic solvent acetonitrile during HPLC, suggesting that it is more hydrophobic than the others. As the predicted mass of active VesB with its signal peptide and propeptide removed, but with an intact C-terminus would be 39.7 kDa, these masses are consistent with rhombosortase cleaving off part of the C-terminus of VesB. The two smallest species of 37,185 Da and 37,027 Da (marked with asterisks in Fig 4A; left panel) matched within +/- 0.01% mass accuracy to molecular species that have either 23 or 25 aa removed from the C-terminus. This represents cleavage of VesB at either Ser_378_Ala_379_ or Ser_380_Ser_381_ immediately upstream of the GlyGly-CTERM domain, which starts at Gly_382_ (Fig 4A; left panel). The two larger species with masses 37,583 and 37,429 Da did not match with theoretical cleavage products and may represent VesB with additional posttranslational modifications.

Purified VesB was also subjected to SDS-PAGE, in-gel trypsin digestion, and LC-MS/MS analysis. Peptide mapping gave a 100% sequence coverage between residues Arg_32_ and Ser_382_. MS/MS analysis revealed a ragged C-terminus of VesB (Fig 4B), consistent with the results from the intact mass analysis described above and with VesB being subject to C-terminal proteolysis. As expected, no peptides containing the intact GlyGly-CTERM were observed. Interestingly, spectra representing modified forms of the C-terminal peptide, IQLDTSPFAPSASSGG, were present. The fragmentation pattern for this peptide showed modification of the C-terminal glycine with either a 43 Da or 197 Da moiety (exemplified in Fig 4C and 4D). The presence of the 43 Da and 197 Da moieties on Gly_383_ generates theoretical intact VesB masses of 37,430 Da and 37,583 Da, respectively; consistent with the major peaks observed in Fig 4A (left panel). Taken together, the two mass spectrometry approaches suggest that VesB primarily undergoes posttranslational modification at Gly_383_. The additional sites of cleavage upstream of the GlyGly-CTERM domain may be the result of proteolysis by extracellular proteases during prolonged growth in LB and/or purification. Support for this suggestion was obtained when VesB was purified at ambient temperature (instead of 4° C) and in the absence of proteinase inhibitors, which yielded the 37,027 Da form of VesB as the dominating species (S1 Fig).

Next, we extracted and purified active cell-associated VesB following growth in M9 medium, which maximizes the yield of the membrane bound form of VesB. While treatment of cells with high salt (2M NaCl), glycine buffer (pH 2.5) or carbonate buffer (pH 11) did not result in appreciable amount of VesB extraction, the non-ionic detergent Triton X-100 was capable of efficiently removing VesB from *V*. *cholerae* cells suggesting that VesB may be associated with cells via protein-lipid or lipid-lipid interaction. We extracted the membrane bound form of VesB with Triton X-100, purified it by benzamidine affinity chromatography and subjected it to SDS-PAGE, in-gel trypsin digestion and LC-MC/MS analysis. Again, we detected two species of the C-terminal peptide IQLDTSPFAPSASSGG with either a 43 Da or 197 Da modification of the terminal Gly_383_ (Fig 4E and 4F).

**Fig 5:**
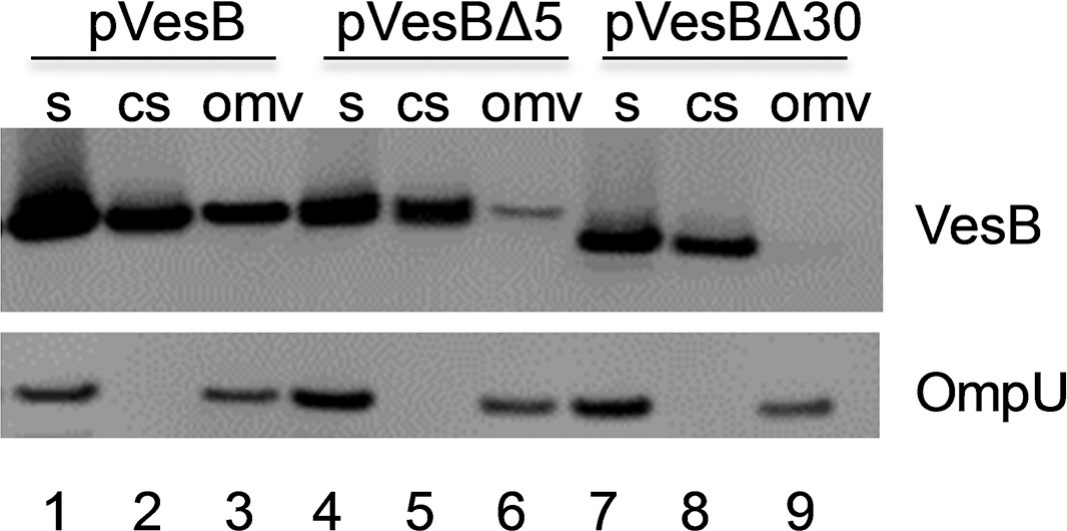
VesB association with OMVs. Supernatants containing VesB, VesBΔ5 and VesBΔ30 were centrifuged at 200,000 x g for 3 hours to pellet OMVs. Supernatant (s), cleared supernatant (cs) and resuspended outer membrane vesicles (omv) were analyzed by SDS-PAGE and immunoblotting with antibodies recognizing VesB and the outer membrane protein OmpU.

The 43 and 197 Da modifications of the terminal Gly_383_ are consistent with ethanolamine (61 Da minus the loss of a water molecule) and glycerophosphoethanolamine (215 Da minus the loss of H_2_O), suggesting that VesB is attached to a phosphoethanolamine moiety (Fig 4G). A peptide with an intact phospholipid such as phosphatidylethanolamine was not identified possibly due to unwanted fragmentation in the mass spectrometer. Nevertheless, the 38,086 Da species detected by intact mass analysis of extracellular VesB (Fig 4A; right panel) is consistent with VesB being modified with phosphatidylethanolamine containing C16 and C18 fatty acids at Gly_383_. The presence of a modified VesB species containing an intact phosphatidylethanolamine among extracellularly released VesB may be due to VesB’s association with outer membrane vesicles (OMVs), a finding recently reported by Mekalanos and colleagues [36]. To test this, we subjected filtered culture supernatant from overnight cultures of *V*. *cholerae* overexpressing VesB to high speed centrifugation, SDS-PAGE and immunoblot analysis. In addition to the major outer membrane protein OmpU, VesB was detected in the pellet (Fig 5, lane 3), suggesting that a fraction of extracellular VesB is pelletable and likely associated with OMVs or OM fragments.

### The presence and subsequent removal of the GlyGly-CTERM domain are essential for cell surface localization of VesB

**Fig 6:**
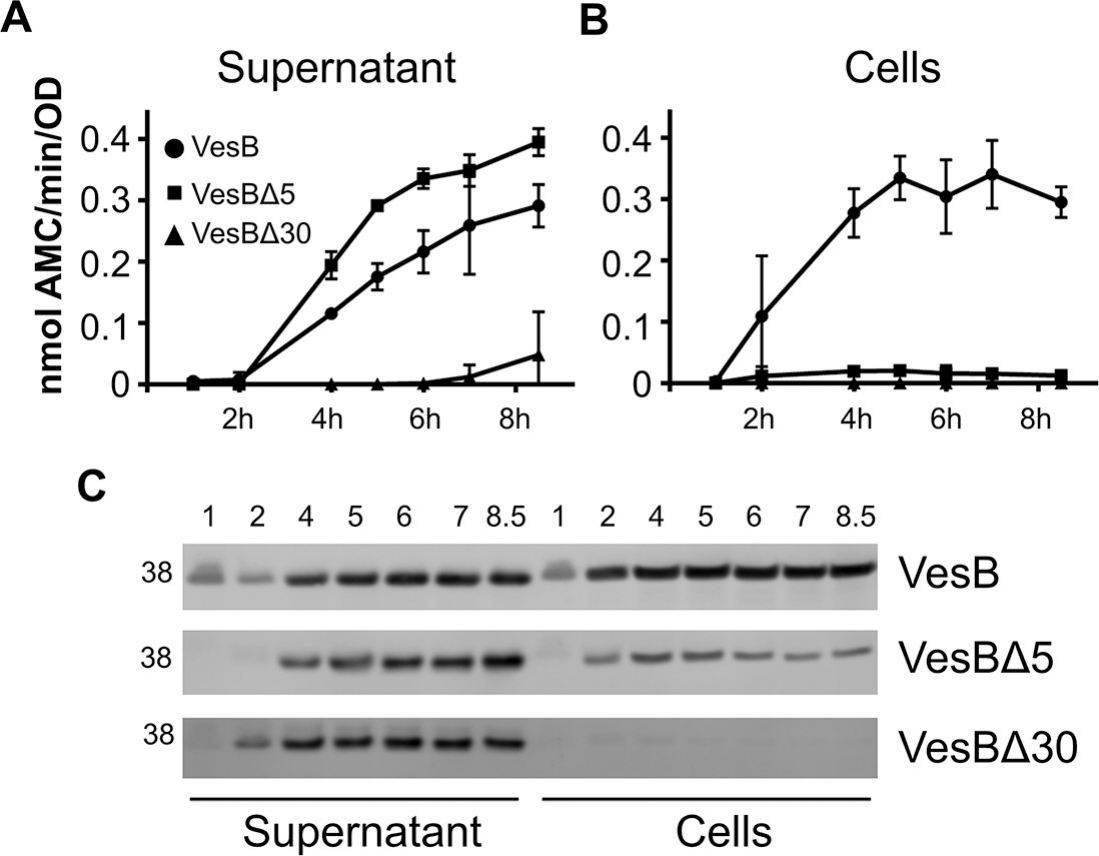
VesB is not localized to the cell surface when produced without the GlyGly-CTERM domain. Cultures of indicated strains expressing VesB or C-terminally deleted VesBΔ5 and VesBΔ30, were grown in LB broth with 100 μg/mL carbenicillin and 40 μM IPTG. Samples were collected at time points indicated, separated into culture supernatant and cells and analyzed for VesB activity and localization. The protease activity assays were performed on samples from three independent experiments and each sample was analyzed in technical triplicates. Bars represent mean ± S.E. p values were generated by comparing culture supernatants and cells containing VesB to those having VesBΔ5 and VesBΔ30. These were all statistically significant at the 4–8.5 hour time points in both supernatants and cells, except for VesB and VesBΔ5 at the 7 hour supernatant time point. A. Using the fluorogenic peptide, Boc-Gln-Ala-Arg-AMC, the activity of VesB, VesBΔ5 and VesBΔ30 was measured in culture supernatants. B. The activity of cell-associated VesB, VesBΔ5 and VesBΔ30 was measured in intact cell suspensions as in A. C. The culture supernatants and cells were subjected to SDS-PAGE and immunoblot analysis using VesB antibodies.

To further address the importance of the GlyGly-CTERM extension, we determined the functional consequences of expressing VesB without an intact GlyGly-CTERM domain. Two VesB variants, VesBΔ5 and VesBΔ30, were constructed and their subcellular location and activity were examined. VesBΔ5 is lacking the C-terminal five residues including the positively charged residues and VesBΔ30 has the additional Gly/Ser rich motif and hydrophobic region removed. These constructs and WT VesB were expressed in Δ*vesABC* cells and at various time points, culture supernatants and cells were collected and analyzed for localization and activity. While VesBΔ5 displayed a slightly higher activity than VesB in the culture supernatant (Fig 6A), no cell-associated activity could be detected (Fig 6B). VesBΔ30 activity was only observed in the 8.5h culture supernatant sample (Fig 6A). The immunoblots correlated well with the activity assays, in that VesB was present in the culture supernatant and cells, while VesBΔ5 was found mostly in the supernatant with a small amount cell-associated. The total yield of VesBΔ30 was lower and this truncated form of VesB was detected exclusively in the supernatant (Fig 6C). The change in subcellular distribution of VesBΔ5 andVesBΔ30 suggests that the presence and the subsequent removal of the GlyGly-CTERM domain by rhombosortase are essential for the cell surface retention of VesB. Consistent with the finding that VesBΔ5 and VesBΔ30 are primarily released from the cell was the finding that little to none of these truncated proteins were associated with OMVs (Fig 5; lanes 6 and 9). In summary, the GlyGly-CTERM domain and processing by rhombosortase are prerequisites for surface localization of VesB.

### Processing of GlyGly-CTERM when fused to EtxB

**Fig 7:**
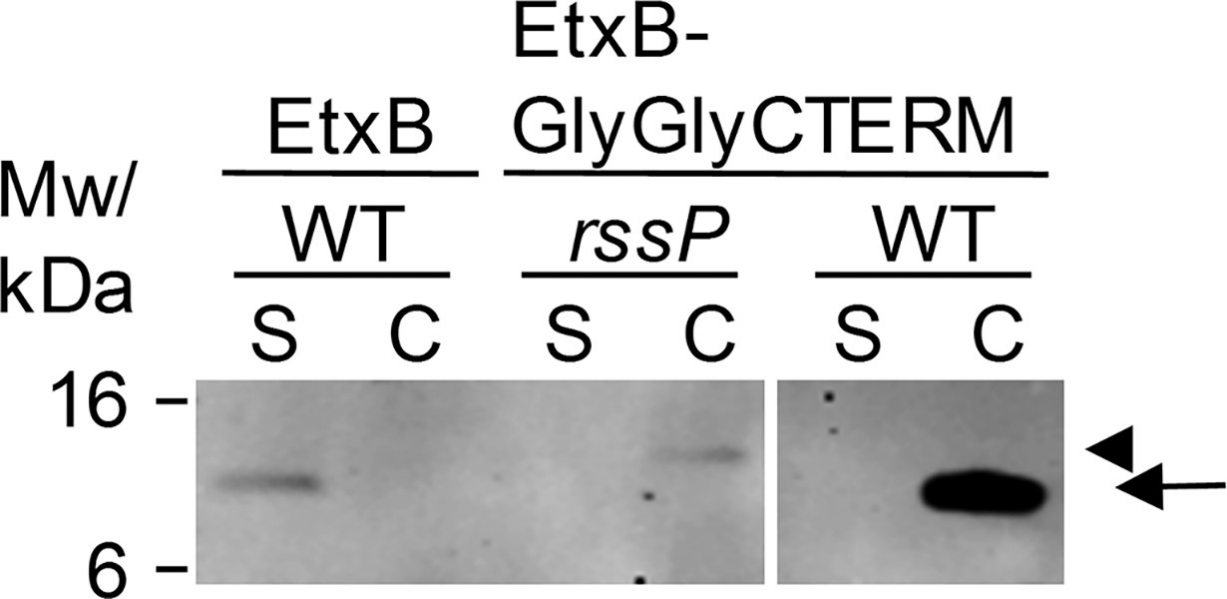
EtxB expressed as a fusion protein with GlyGly-CTERM. Cultures of WT and *rssP*::*kan* mutant strains expressing EtxB or EtxB-GlyGly-CTERM were grown overnight in LB broth with 100 μg/mL carbenicillin and 50 μM IPTG. Cells (C) and culture supernatants (S) were subjected to SDS-PAGE and immunoblotting with monoclonal anti-EtxB. The positions of intact EtxB-GlyGly-CTERM (arrowhead) and EtxB and processed EtxB-GlyGly-CTERM (arrow) are indicated on the right.

To further address the requirement for processing by rhombosortase, we fused the GlyGly-CTERM domain from VesB to the C-terminus of the *E*. *coli* heat-labile enterotoxin B subunit, EtxB. While EtxB, a homolog of the cholera toxin B subunit, was secreted by *V*. *cholerae* as has been shown previously [6, 37], EtxB-GlyGly-CTERM remained associated with the cells (Fig 7). When expressed in the *rssP*::*kan* mutant, EtxB-GlyGly-CTERM was barely detected, but appeared to be larger in size, suggesting that it may retain the GlyGly-CTERM domain in the absence of rhombosortase. This result suggests that EtxB-GlyGly-CTERM is recognized and processed by rhombosortase; however, whether fusing a GlyGly-CTERM domain to any protein with an N-terminal signal peptide will result in a hybrid protein that is targeted by rhombosortase needs to be addressed in much greater detail.

## Discussion

In this study, we used the trypsin-like serine protease VesB as a model protein to determine the relationship between the GlyGly-CTERM domain, rhombosortase, and the T2SS in *V*. *cholerae*. T2S substrates translocate through the outer membrane and are either released into the extracellular milieu or retained on the cell surface through a variety of mechanisms. We show here that the majority of VesB is surface-associated; however, depending on growth conditions, various amounts of the surface-localized VesB are released to the extracellular space. Rhombosortase cleaves off the GlyGly-CTERM domain and the newly generated C-terminus is further modified to retain VesB on the cell surface once transported through the T2SS. When rhombosortase is absent, full-length VesB is largely subjected to degradation, but a small fraction is cleaved by GlpG. However, there is likely no additional posttranslational modification of GlpG-cleaved VesB and, therefore, GlpG-cleaved VesB is released to the extracellular milieu. Additionally, rhombosortase-mediated C-terminal processing leads to subsequent VesB auto-activation, while cleavage by GlpG in the absence of rhombosortase results in an inactive VesB, indicating that either correct C-terminal processing of VesB and/or its localization to the outer membrane are required for its auto-activation.

**Fig 8:**
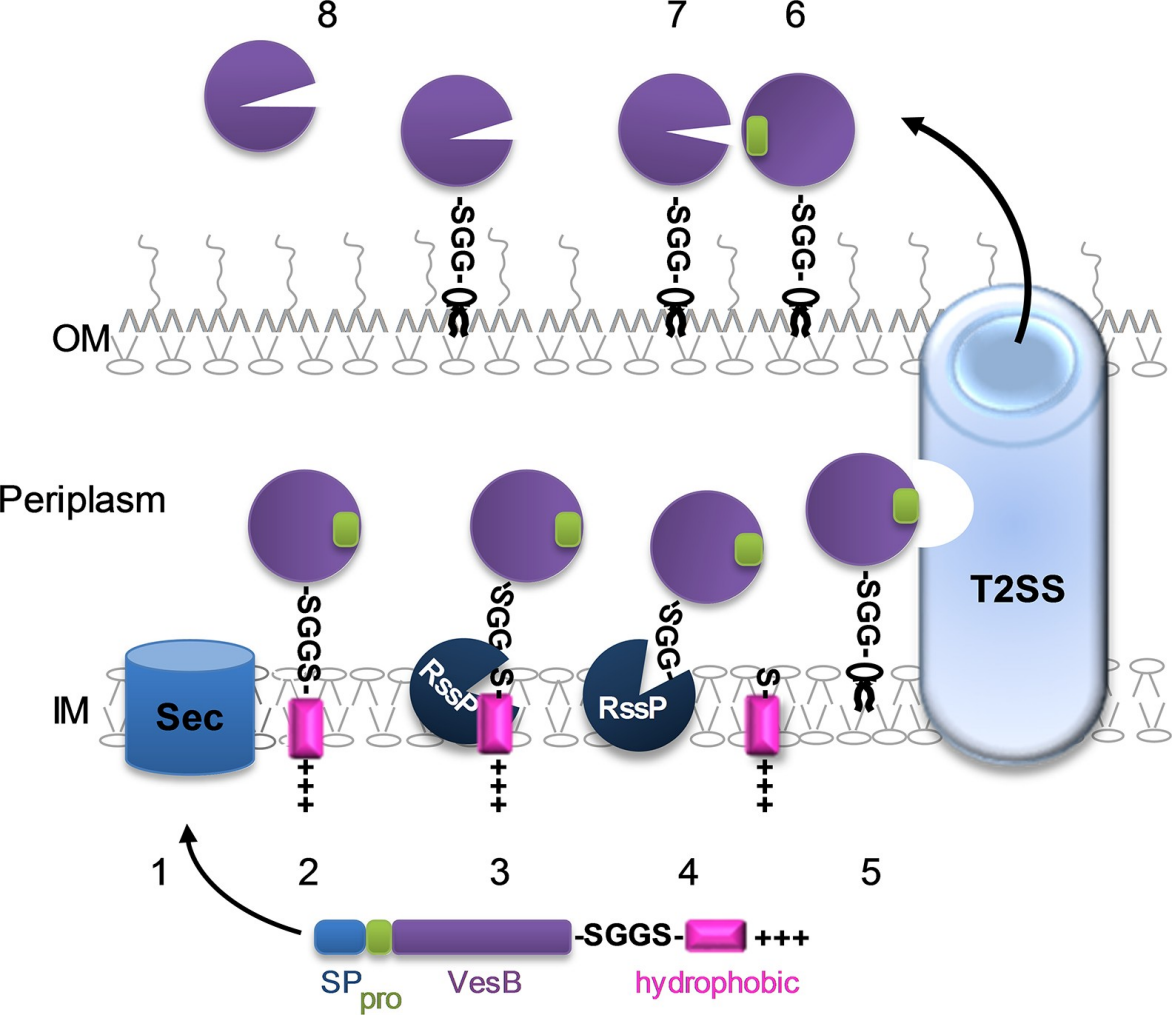
Model. VesB (purple) is produced in the cytoplasm with an N-terminal signal peptide (blue), propeptide (green), a hydrophobic domain (magenta) and a positively charged C-terminus (+++). VesB is directed to the Sec machinery by the signal peptide (1). As the protein is pulled into the periplasm via the Sec system, the signal peptide is cleaved off, the GlyGly-CTERM domain is positioned in the inner membrane (IM), and the protein folds (2). Rhombosortase (RssP; dark blue) recognizes and cleaves the GlyGly-CTERM domain (3). The acyl enzyme intermediate is resolved by either H_2_0 or ethanolamine and the newly generated C-terminus of VesB is conjugated to a glycerophosphoethanolamine component, possibly phosphatidylethanolamine (4). VesB is recognized by the T2SS (5) and transported to the cell surface (6) where its propeptide is removed in *trans* by a neighboring, active VesB (7). A fraction of surface-associated VesB is released through OMV blebbing and additional processing protease(s) (8).

Taking all of the data together, we have assembled a model for successful delivery of VesB to the cell surface of *V*. *cholerae* that involves its GlyGly-CTERM domain, rhombosortase and the T2SS (Fig 8). VesB is synthesized in the cytoplasm with an N-terminal signal peptide and a C-terminal GlyGly-CTERM domain. VesB enters the Sec system via the signal peptide and, as the protein is translocated through the Sec system, the GlyGly-CTERM domain is positioned in the inner membrane. The signal peptide is cleaved off and VesB folds. Rhombosortase cleaves the GlyGly-CTERM domain and possibly further modifies the newly generated VesB C-terminus by attaching it to a glycerophosphoethanolamine containing lipid (possibly phosphatidylethanolamine) via transamidation. It is also possible that a second enzyme is responsible for this posttranslational modification event; however, we have no evidence for a separate transamidase enzyme at this time. VesB is then translocated from the inner membrane by the T2SS and delivered to the cell surface. Once at the cell surface, we speculate that the propeptide is removed in *trans* by a nearby active VesB resulting in auto-activation. Under some growth conditions, surface-localized VesB may then be released through outer membrane blebbing and/or detachment by extracellular protease(s).

The rhombosortase/GlyGly-CTERM system may be compared to the sortase/LPXTG system of Gram-positive organisms and the archaeosortase/PGF-CTERM system of archaea. In all three systems, substrates contain a C-terminal tripartite domain consisting of a recognition motif (GG, LPXTG or PGF) followed by a hydrophobic region and positively charged residues [38, 39]. For example, in *Staphylococcus aureus*, sortase cleaves staphylococcal protein A (SPA), a LPXTG substrate, between the Thr and Gly residues, resulting in an acyl-enzyme intermediate that is resolved by lipid II, a precursor of peptidoglycan (PG), instead of H_2_O [40]. This transamidation process results in PG-anchoring of SPA [40]. VesB may also undergo C-terminal transamidation rather than complete hydrolysis, where the terminal NH_2_ group of an ethanolamine-containing component serves as the attacking nucleophile instead of H_2_O, which is scarce in the membrane environment. The detection of a species containing a 197-Da modification of the C-terminal glycine in VesB is consistent with a glycerophosphoethanolamine modification. Because extraction of VesB from the surface of *V*. *cholerae* requires a detergent, it is possible that VesB is attached to a glycerophosphoethanolamine containing lipid, possibly phosphatidylethanolamine generating a size of VesB that is consistent with the protein peak in Fig 4A; right panel. While this is the first time that this type of posttranslational modification is described in prokaryotes, Atg8, a ubiquitin-like protein required for autophagosome formation in yeast, is cleaved at the C-terminus by a cysteine protease to expose a glycine that is subsequently attached to phosphatidylethanolamine (PE) via amidation [41, 42]. When Atg8-PE was analyzed by mass spectrometry a C-terminal 197 Da glycerophosphoethanolamine moiety was also detected at a C-terminal glycine. There is also some resemblance between surface localization of VesB and glycosylphosphatidylinositol (GPI)-anchored proteins in eukaryotes [43], as both processes involve the removal of a trans-membrane domain and attachment of the cleaved protein to a lipid via a phosphoethanolamine linker. Further studies are planned to elucidate the mechanism of C-terminal modification of VesB and to determine whether rhombosortase has transamidase activity. Rhombosortase does form a distinct group with other rhombosortases within the greater rhomboid protease family. Rhombosortase is only 23% identical to *E*. *coli* GlpG and it is lacking the 90 amino acid N-terminal cytoplasmic domain, whose function has yet to be determined. This globular domain is present in most rhomboid proteases except *Haemophilus influenzae* GlpG and *P*. *stuartii* AarA, and in the mitochondrial PARL proteases it is replaced by a transmembrane domain [24, 44]. It is possible that sequence differences and the lack of the cytoplasmic domain favor transamidation over hydrolysis by rhombosortase. For example, the active site of rhombosortase may be less exposed to water than that of rhomboid protease. As a consequence, the transient acyl-enzyme intermediate, formed when the serine hydroxy group of rhombosortase attaches to the acyl moiety of VesB, will react with the nucleophilic NH2 group of ethanolamine instead of water [45].

Under some conditions, we observe more VesB in the supernatant than others (for example, in LB vs. M9 medium containing casamino acids and glucose). Similar findings have been reported for other surface-attached proteins. For example, it was recently shown that the cell wall-attached sortase substrate SPA is released into the culture supernatant by murein hydrolases [46]. Additionally, GPI-anchored proteins, like prostasin, can be released from the cell surface by lipid-targeting enzymes like phospholipase C [47]. Surface-localized VesB could similarly undergo processing by an enzyme such as an extracellular protease that removes it from the cell surface. Our results suggest that release of VesB may also occur via OM blebbing.

The dual locations of VesB pose an interesting question of why a fraction of the protein is released to the extracellular space. While it is possible that the release of surface-localized VesB is a laboratory-induced artifact, dual locations have also been reported for the GlyGly-CTERM containing protein ExeM required for biofilm formation by *Shewanella oneidensis*. While ExeM has been found as an active nuclease in the culture supernatant of *S*. *oneidensis* [48], other studies involving proteomic analysis of membranes have identified ExeM as a membrane-associated protein [49, 50]. *V*. *cholerae* Xds, an ExeM homolog, displays a similar distribution, as Xds nuclease activity has been detected in both culture supernatant and in association with cells [17, 51]. VesB and other GlyGly-CTERM proteins, which are mostly hydrolytic enzymes, may be of benefit to the individual bacterium when retained on the surface where they can break down macromolecules and generate nutrients such as short peptides for immediate cellular uptake. On the other hand, in the context of a biofilm, the released form of these proteins could benefit the community at large.

In summary, VesB has a novel GlyGly-CTERM domain and utilizes rhombosortase and the T2SS to be correctly processed, translocated across the cell envelope, and retained on the cell surface of *V*. *cholerae*. The rhombosortase/GlyGly-CTERM system offers a new alternative method of surface association of T2S substrates that differs from previously observed mechanisms. PnlH from *D*. *dadantii* possesses a non-cleavable TAT specific signal peptide that is needed for its outer membrane retention [52], heat-labile enterotoxin from ETEC is localized on the cell surface via an interaction with lipopolysaccharides [53] and the lipidated N-terminus of pullulanase keeps it surface-associated in *K*. *oxytoca* through an unknown mechanism [54]. Furthermore, the newly acquired knowledge of the GlyGly-CTERM domain of VesB provides insight to the subcellular distribution of ExeM and Xds from *S*. *oneidensis* and *V*. *cholerae*, respectively. Based on our findings, a large fraction of these proteins are also likely retained on the cell surface in a process that involves their GlyGly-CTERM domains and rhombosortase [17, 48–51]. Our findings may also provide insight into the mechanism of C-terminal processing of the PGF-CTERM domain of archaeal S-layer glycoprotein by archaeosortase. While the S-layer glycoprotein is lipid modified and can be extracted with Triton X-100, it is not known where archaeosortase cleaves and the site of lipid modification has not yet been identified [38].

## Materials and methods

### Bacterial strains and plasmids

The *V*. *cholerae* El Tor O1 strain, N16961, and the isogenic Δ*eps* [37], Δ*epsD* [55], Δ*vesB* and Δ*vesABC* [10] mutants were used in this study. All plasmids and primers are listed in Table 2. All polymerase chain reactions (PCR), cloning and restriction enzyme digestions were done with Phusion Polymerase, T4 DNA ligase and restriction enzymes from New England Biolabs and primers that were synthesized at IDT Technologies. pCRScript (Stratagene) and pMMB67EH constructs were transformed into *E*.*coli* MC1061 and pCVD442 constructs into SY327λpir. Triparental conjugation was performed with a helper strain, MM294/pRK2013 to transfer plasmids into N16961 and its isogenic mutants [56].

**Table 2:** List of primers and plasmids used.

Gene or Plasmids	Genotypes	Forward Primer 5' to 3'	Reverse Primer 5' to 3'	Relevant Restriction Enzymes sites
pCRscrpt	Cloning vector, Amp^R^			
pMMB67EH	*Ptac promoter*, *amp*^*R*^			
pCVD442	*ori R6K mobRP4 sacB* Amp^R^			
pK18mobsacB	*ori-pMB1 oriT* (RP4) *sacB lacZ* Kan			
*rssP*		GCGGGATCCGAGTGGGCGCTTTTGTTC	TCCTGTTTATTGCGATAGTTTACG	SacI and SalI
*kan*		GCGTGATCACCGGAATTGCCAGCT	CGCTGATCATCAGAAGAACTCGTC	BclI
*glpG—upstream fragment*		GCGTCTAGATACATATTGTGGATCGAAAATCAC	AAATGCTAGTCCTTAACTCGCTTCGATGGG	SacI
*glpG—downstream fragment*		GGACTAGCATTTCCTCCTTTCTTGCGCCGC	CGCCCCGGGTGGTTCTGGCTGTAATGCCGTCGT	SalI
*vesBΔ5*		[57]	CGCCTGCAGTCACATCCACAATGGAGCCAAAA	EcoRI and PstI
*vesBΔ30*		[57]	CGCCTGCAGTCACGAAGTATCCAGTTGAATACGA	EcoRI and PstI
*vesB-S221A*		TCATGTCAGGGAGATGCTGGTGGCCCAATTGTA	TACAATTGGGCCACCAGCATCTCCCTGACATGA	
*rssP-S102A - upstream*		GAAATGAGCTGTTGACAATTAATC	CAGTCTTATGTTGGGCTCGCCAGCAGTTGG	SacI
*rssP-S102A - downstream*		GTTGGGCTCGCCGGCACCTTGCATGGTCTG	CTGTTTTATCAGACCGCTTCTGCG	SalI
*rssP-H160A - upstream*		GAAATGAGCTGTTGACAATTAATC	CGAGTAGCGACGGAAGCCGCTTTGGCCGGT	SacI
*rssP-H160A - downstream*		ACGGAAGCCGCTTTGGCCGGTTTAGTCGGC	CTGTTTTATCAGACCGCTTCTGCG	SalI
*etxB-glyglyCterm-* *upstream*		GAAATGAGCTGTTGACAATTAATC	GCCAGAAGAGGCACTGCCGTTTTCCATACT	
*etxB-glyglyCterm-* *downstream*		AACGGCAGTGCCTCTTCTGGCGGTTCCATC	CTGTTTTATCAGACCGCTTCTGCG	

A kanamycin insertional rhombosortase mutant (*rssP*::*kan)* was created by amplifying the rhombosortase (*rssP*) gene (VC1981) from *V*. *cholerae* N16961 chromosomal DNA using rssP primers. The fragment was ligated into a high copy plasmid, pCRscript. A kanamycin cassette was amplified from pK18mobsacB using kan primers containing *BclI* restriction enzyme sites and cloned into the native *BclI* site in *rssP*. The *rssP*::*kan* fragment was moved into the suicide plasmid, pCVD442. The rhomboid protease gene from *V*.*cholerae*, *glpG* (VC0099) was deleted from the chromosome by amplifying 500 base pair regions upstream and downstream from *glpG* followed by overlap extension PCR of the two fragments to generate a 1.0 kbp fragment that was cloned into pCVD442.

For complementation, *rssP* was amplified and cloned into pMMB67EH, a low copy vector with an IPTG inducible promoter and ampicillin cassette to generate pRssP. Using primers with base pair changes and the pRssP plasmid as template, fragments with *rssP-*S102A and *rssP*-H160A were made and cloned into pMMB67EH. *vesBΔ5* and *vesBΔ30* were amplified from pCRscript carrying *vesB* using newly created reverse primers with the forward primer originally used to clone *vesB* [57]. PCR fragments were cloned into pMMB67EH.

Plasmids pMMB68 [58] and pVesB [57] and primers annealing to the ends of *etxB* and *vesB*, were used to generate EtxB fused to the GlyGly-CTERM extension of VesB.

### Growth conditions

Strains were either grown on Luria-Bertani (LB) agar/broth (Fisher) or M9 media containing 0.4% glucose and 0.4% casamino acids with 100 μg/mL of carbenicillin (Sigma) when plasmids were present.

### Triton X-100 extraction and purification of cell-associated VesB

Δ*vesABC*/pVesB was grown in the presence of 50 μM IPTG in M9 media supplemented with glucose and casamino acids for 4 h. Following the removal of culture supernatant, cells were resuspended and sonicated. After removing unlysed cells, the cell envelope was pelleted at 170,000 x g for 1h. Membrane pellet was resuspended in 50 mM Tris-HCl pH 8.0/450 mM NaCl buffer containing 2% Triton X-100. Following another high speed centrifugation step, Triton X-100 soluble VesB was purified by benzamidine affinity chromatography [11] in the presence of 2% Triton X-100. Following washing with the above buffer containing Triton X-100, VesB was eluted with 10 mM benzamidine in the presence of Triton X-100. Fractions containing purified VesB were collected, pooled precipitated with pyrogallol red-molybdate-methanol as described [10], and subjected to SDS-PAGE, in-gel trypsin digestion and LC-MS/MS analysis as described below.

#### Isolation of OMVs

Culture supernatants isolated from *V*. *cholerae* cultures were centrifuged at 200,000 x g for 3 h to pellet OMVs. The OMVs, the starting material representing unfractionated culture supernatants, and cleared supernatants were analyzed by SDS-PAGE and immunoblotting with antibodies directed against VesB or OmpU.

### Sodium dodecyl sulfate-polyacrylamide gel electrophoresis (SDS-PAGE) and immunoblotting

Samples were prepared and analyzed by SDS-PAGE and immunoblotting as described previously [37]. Polyclonal antiserum against VesB [11] was incubated with culture supernatant from the Δ*vesABC* mutant for 1 h to pre-absorb cross-reactive antibodies prior to incubating with the nitrocellulose membrane for 2 h (1:5,000). OmpU antibodies (gift from K. Klose) were used at 1:20,000 and incubated for 2 h and monoclonal EtxB antibody was used at 1:30,000 [37]. Horseradish peroxidase-conjugated goat anti-rabbit immunoglobulin G (Bio-Rad) used at 1:20,000 was incubated with the membrane for 1 h. Membranes were developed using ECL 2 Western blotting reagent (Thermo Fisher) and visualized using a Typhoon Trio variable mode imager system and Image Quant software.

### Protease assay

*V*. *cholerae* supernatants and whole cells were measured for protease activity using *N-tert*-butoxycarbonyl-Gln-Ala-Arg-7-amido-4-methylcoumarin as described previously [11]. Change in fluorescence per minute was calculated and converted to moles of methlycoumarin (AMC) cleaved per minute via a standard curve with known concentrations of AMC. The rate of AMC generation was normalized by OD_600_ of the cultures.

### Cell labeling and microscopy

Cells were washed, blocked with 2% BSA and incubated with 1:1000 of VesB antiserum that was pre-incubated with Δ*vesABC* cells to remove cross-reactive antibodies. Following incubation with 1:1000 of Alexa Fluor 488 F(ab′)_2_ goat anti-rabbit immunoglobulin G (Invitrogen) and washing, fluorescence was measured (Ex 495 nm/ Em 519 nm). The cells were also visualized by differential interference contrast and fluorescent microscopy using a Nikon Eclipse 90i fluorescence microscope equipped with a Nikon Plan Apo VC 100× (1.4 numerical aperture) oil immersion objective and a CoolSNAP_HQ_^2^ digital camera as previously described [57].

### Intact mass determination of VesB

Intact mass analysis of VesB (10 μl of a 20-μM solution in 10 mM Tris-HCl), purified from *V*. *cholerae* culture supernatant as described [11], was conducted on an Agilent 6224 ESI-TOF mass spectrometer in conjunction with an Agilent 1260 Infinity binary pump HPLC system as follows: mobile phase A, 0.1% formic acid in water; mobile phase B, 0.1% formic acid in 90% acetonitrile/10% water; gradient, 20% B to 90% B in 15 min; flow rate, 200 μl/min; positive ion detection; profile mode; mass range, *m/z* 100– *m/z* 3,200; fragmentor, 225 V; skimmer, 65 V; capillary voltage, 4,000 V; gas temperature, 325°C. The maximum entropy deconvolution algorithm in MassHunter BioConfirm (Agilent) was used for molecular mass determination of components in the sample.

### Proteomics/GeLC-MS/MS analysis

Culture supernatants of WT and *rssP*::*kan* strains were prepared as described with the following modifications [59]. Culture supernatants from three independent experiments were combined and 20 μg of protein was processed by SDS-PAGE. The gel was stained with InstantBlue (Expedeon) and excised into ten equal sized segments. Gel segments were digested with a ProGest robot (DigiLab) with the following protocol: washed with 25mM ammonium bicarbonate followed by acetonitrile, reduced with 10mM dithiothreitol at 60°C followed by alkylation with 50mM iodoacetamide at RT, digested with trypsin (Promega) at 37°C for 4h, quenched with formic acid, and the supernatant was analyzed directly without further processing. Each gel digest was analyzed by nano LC/MS/MS with a Waters NanoAcquity HPLC system interfaced to a ThermoFisher LTQ Orbitrap Velos Pro. Peptides were loaded on a trapping column and eluted over a 75μm analytical column at 350nL/min; both columns were packed with Jupiter Proteo resin (Phenomenex). The mass spectrometer was operated in data-dependent mode, with MS performed in the Orbitrap at 60,000 FWHM resolution and MS/MS performed in the LTQ. The fifteen most abundant ions were selected for MS/MS. Data were searched using a local copy of Mascot (Matrix Science, UK) with the following parameters: Enzyme: Trypsin; Database: Swissprot *V*. *cholerae* El Tor N16961 (concatenated forward and reverse plus common contaminants); Fixed modifications: Carbamidomethyl (C); Variable modifications: Oxidation (M), Acetyl (N-term), Pyro-Glu (N-term Q), Deamidation (N,Q); ethanolamine (C-term), glycerophosphoethanolamine (C-term) and carbamyl (C-term). Mascot DAT files were processed in Scaffold (Proteome Software Inc.) for determination of protein and peptide probabilities. Data were filtered using protein and peptide thresholds of 99% and 95%, respectively, and requiring at least two unique peptides per protein. The complete mass spectrometry proteomics data have been deposited to the ProteomeXchange Consortium (http://proteomecentral.proteomexchange.org) via the PRIDE partner repository [60] with the dataset identifier PXD000896. Data for the six GlyGly-CTERM proteins are shown in Table 1.

In-gel trypsin digested VesB, purified from culture supernatant or Triton X-100 extracted membranes of *V*. *cholerae*, was analyzed by nano LC/MS/MS with a Proxeon EASY-nLC 1000 HPLC system interfaced to a ThermoFisher Q Exactive mass spectrometer. Data were searched using a local copy of Mascot and subsequently processed in Scaffold with the parameters described above. Additional Mascot searches were performed with the enzyme specified as “no enzyme” instead of trypsin, in order to search for truncated C-terminal peptides The data were deposited to the ProteomeXchange Consortium with the dataset identifier PXD003261.

### Statistical analysis

Statistical significance between WT and mutant samples was assessed by Student’s T-test. Results yielding a P value of <0.05 were considered statistically significant.

## Supporting information

S1 Fig Mass analysis of VesB. VesB was overexpressed and purified from supernatants of LB-grown *V*. *cholerae* culture at ambient temperature in the absence of protease inhibitors. The purified material was subjected to intact mass analysis using an Agilent 6224 ESI-TOF mass spectrometer. Deconvoluted ESI mass spectrum indicates a major VesB species. Accuracy of the instrument: 0.01% of molecular mass.
(PDF)
