# Supplementary material for: C-terminal processing of GlyGly-CTERM containing proteins by rhombosortase in Vibrio cholerae
Source: PLoS Pathog. 2018 Oct 23;14(10):e1007341. doi: 10.1371/journal.ppat.1007341 (PMC6219818; doi:10.1371/journal.ppat.1007341)
Supplement: S1 Fig — VesB was overexpressed and purified from supernatants of LB-grown V. cholerae culture at ambient temperature in the absence of protease inhibitors. The purified material was subjected to intact mass analysis using an Agilent 6224 ESI-TOF mass spectrometer. Deconvoluted ESI mass spectrum indicates a major VesB species. Accuracy of the instrument: 0.01% of molecular mass. (PDF) [file ppat.1007341.s001.pdf]

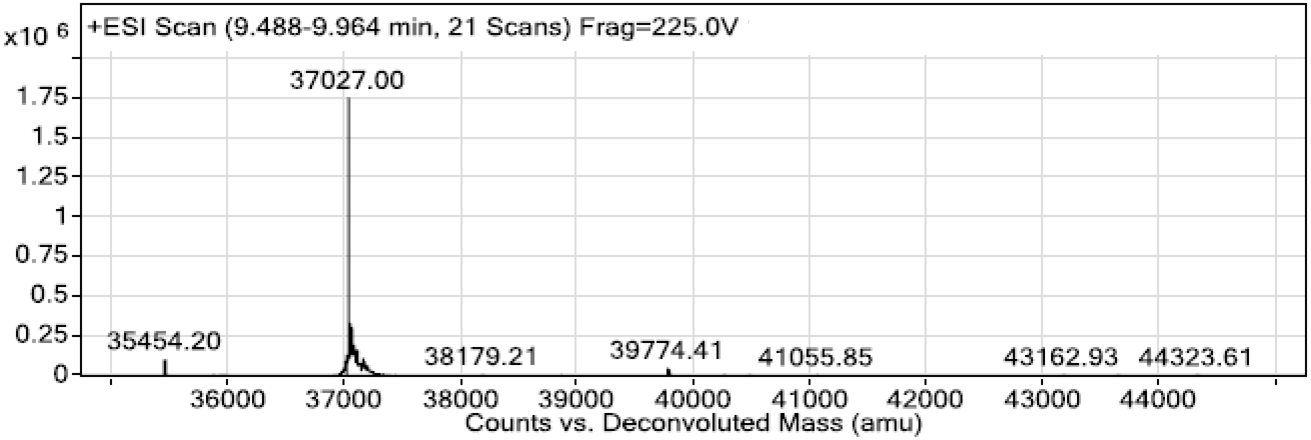

Figure S1. Mass analysis of VesB. VesB was overexpressed and purified from supernatants of LB-grown *V. cholerae* culture at ambient temperature in the absence of protease inhibitors. The purified material was subjected to intact mass analysis using an Agilent 6224 ESI-TOF mass spectrometer. Deconvoluted ESI mass spectrum indicates a major VesB species. Accuracy of the instrument: 0.01% of molecular mass.
